# Enhanced nitrogen dynamics and uptake in cotton via organic liquid fertilizer partial substitution

**DOI:** 10.3389/fpls.2026.1781486

**Published:** 2026-05-05

**Authors:** Lianjie Jiang, Jinhu Zhi, Xinlu Bai, Lei Wang, Shengwang Chai, Muhammad Abu Bakar Hayat, Shuyu Zhang, Runze Wang, Sha Xue

**Affiliations:** 1 College of Agriculture, Tarim University, Alar, China; 2 Key Laboratory of Genetic Improvement and Efficient Production of Special Crops in Arid Areas, Tarim University, Alar, China; 3 Research Center of Oasis Agricultural Resources and Environment in Southern, Tarim University, Alar, China; 4 Northwest A&F University Institute of Soil and Water Conservation, Yangling, China

**Keywords:** chemical fertilizer, cotton, nitrogen utilization, organic liquid fertilizer, soil nutrients

## Abstract

Addressing water scarcity and soil degradation in the extremely arid region of the southern Tianshan Mountains in Xinjiang, China, requires reducing chemical fertilizer application while maintaining cotton yields, which is crucial for sustainable agricultural development. Method: This study investigated the regulatory effects of partially replacing chemical fertilizers with organic liquid fertilizers on soil nutrients, plant nitrogen uptake, and yield components in cotton fields through a two-year (2024–2025) field trial. Five treatments were established: no nitrogen fertilizer (CK), 100% urea (T1), organic liquid fertilizer replacing 15% (T2), 30% (T3), and 45% (T4) of urea nitrogen. The 30% organic liquid fertilizer replacement treatment (T3) yielded the highest cottonseed cotton production in both years, at 8,375.3 kg·ha^−1^ and 10,014 kg·ha^−1^, respectively, representing increases of 0.7% and 4.81% compared to the conventional fertilization treatment (T1). This treatment exhibited significantly higher agronomic nitrogen use efficiency (ANUE) and partial productivity of nitrogen fertilizer (PFP) than other fertilization treatments. In 2025, ANUE reached 4.34 kg·kg^−1^, ranking highest among all treatments. Regarding plant nitrogen content, T3 treatment showed significantly higher root and leaf nitrogen levels than other treatments during the PBS stage. Simultaneously, soil nitrogen dynamics analysis indicated that the nitrate nitrogen (NO_3_^−^-N) content in the T3 treatment was significantly higher than that in the T1 treatment, reaching 86.65 mg·kg^−1^ in 2024 and 83.25 mg·kg^−1^ in 2025. Correlation analysis further confirmed that soil nitrate nitrogen content and bolls per plant (NBPT) are key factors influencing seed cotton yield, exhibiting a highly positive correlation. In summary, replacing 30% of chemical fertilizer nitrogen with organic liquid fertilizer nitrogen in drip-irrigated cotton fields not only optimizes soil nitrogen supply but also enhances nutrient use efficiency. This approach achieves dual objectives of increased yield and sustainable production, providing scientific basis for integrated water and fertilizer management in extreme arid regions.

## 1 Introduction

Global climate change has worsened the issue of water scarcity for agriculture in regions susceptible to severe drought (Mousavi et al., 2025), especially in the southern foothills of the Tianshan Mountains in Xinjiang, China. Poor soil quality, lack of organic matter, and impeded nutrient cycling severely limit crop productivity (Abu-Ria et al., 2024). Cotton is a pivotal cash crop in Xinjiang, and it has also been identified as a “pioneer crop” for the development and utilisation of saline-alkali land (Han et al., 2023). In 2023, Xinjiang’s cotton cultivation area and output reached 2470 thousand hectares and 5.39 million tonnes respectively, ranking first in the country and accounting for over 90% of its cotton production. The southern Xinjiang region enjoys abundant sunshine and large temperature differences between day and night, which is conducive to the accumulation of dry matter and the formation of economic yields in cotton (Li et al., 2021). The area planted with cotton in this region accounts for approximately 70% of the total cotton planting area in Xinjiang, making it one of the primary cotton-producing regions in Xinjiang and even the entire country. However, cotton cultivation currently faces serious challenges (Xia et al., 2025). Scholars both domestically and internationally generally agree that excessive use of chemical fertilizers not only affects cotton growth, but also causes soil degradation and nutrient imbalance (Hsu and Lai, 2022; Shi et al., 2024; Yan et al., 2021).To address this issue, China has introduced a series of policies since 2015: the Central Committee’s No. 1 Document made special arrangements for “strengthening agricultural ecological governance”; the Ministry of Agriculture and Rural Affairs’ “Implementation Opinions on Winning the Battle Against Agricultural Non-point Source Pollution” clearly put forward the goal and tasks of “one control, two reductions, and three basics,” and reducing fertilizer use to alleviate environmental pressure is an important part of this work. Reducing fertilizer use and replacing it with organic fertilizer are good solutions.

Xinjiang has a wide variety and large quantity of organic fertilizers, with abundant resources. However, the vast majority of organic waste is directly discharged into the environment without treatment, with only 10-20% being treated or utilized. Although organic fertilizers contain rich organic matter and various nutrients, their nutrients are released slowly and their content is low, making it difficult to meet the high yield requirements of crops (Wang et al., 2024a). Extensive testing confirms (Gao et al., 2023; Sun et al., 2023; Tounkara et al., 2020), that the combined application of organic and inorganic fertilizers can effectively enrich the soil and improve soil fertility (Ndung'u et al., 2021; Zhu et al., 2025), which is an important source of achieving high crop yields and efficient fertilizer utilization (Anyega et al., 2021). This application method can coordinate the supply of fast-acting and slow-acting nutrients, significantly improving nutrient utilization efficiency (Ning et al., 2022). Specifically, the combined application of organic and inorganic fertilizers can improve soil physical and chemical properties (Liu et al., 2024), also increase the availability of soil nutrients and organic matter content (Wang et al., 2024b), thereby reducing fertilizer loss and enhancing soil fertility and ecosystem productivity (Bhardwaj et al., 2014). Compared with the use of chemical fertilizers alone, organic fertilizers can reduce soil bulk density and increase porosity, aggregate structure, and plow layer structure (Lin et al., 2022; Liu et al., 2025; Wang et al., 2023a). As fertilizer application rates increase, the nitrogen uptake and yield of organic fertilizers partially replacing inorganic fertilizers also show a significant upward trend (Wang et al., 2023a). This has notably increased the content of mineral nitrogen in the soil, improved nitrogen supply, and enhanced nitrogen fertilizer utilization efficiency (Farooq et al., 2024).

The advent of organic liquid fertilizer, a recent innovation, has introduced a novel method of fertilizer application, namely through drip irrigation systems. In the face of the mounting scarcity of water resources, the cotton industry in southern Xinjiang has adopted two primary cultivation methods: sub-mulch drip irrigation and dry sowing, wet emergence (Liang et al., 2025). Conventional techniques for the application of organic fertilizers are no longer adequate for these novel cultivation models. Conversely, the use of OLF has been demonstrated to be efficacious in enhancing soil organic matter while concomitantly facilitating the integration of water and fertilizer application (Shi et al., 2021). Current research indicates that humic acid liquid fertilizer (Deng et al., 2023), amino acid liquid fertilizer (Malik et al., 2022), and microbial fermentation liquid can promote crop growth and improve the soil environment in both greenhouse crops and field crops (Iftikhar et al., 2019). However, there are few reports on their application in cotton, an economic crop in extremely arid regions. Therefore, investigating the impact of different proportions of organic liquid fertilizer replacing chemical fertilizers on soil nutrients in oasis cotton fields is of great significance, as it can provide a scientific basis for sustainable cotton cultivation and fertilizer reduction in extremely arid areas.

## 2 Materials and methods

### 2.1 Experimental site

**Figure 1:**
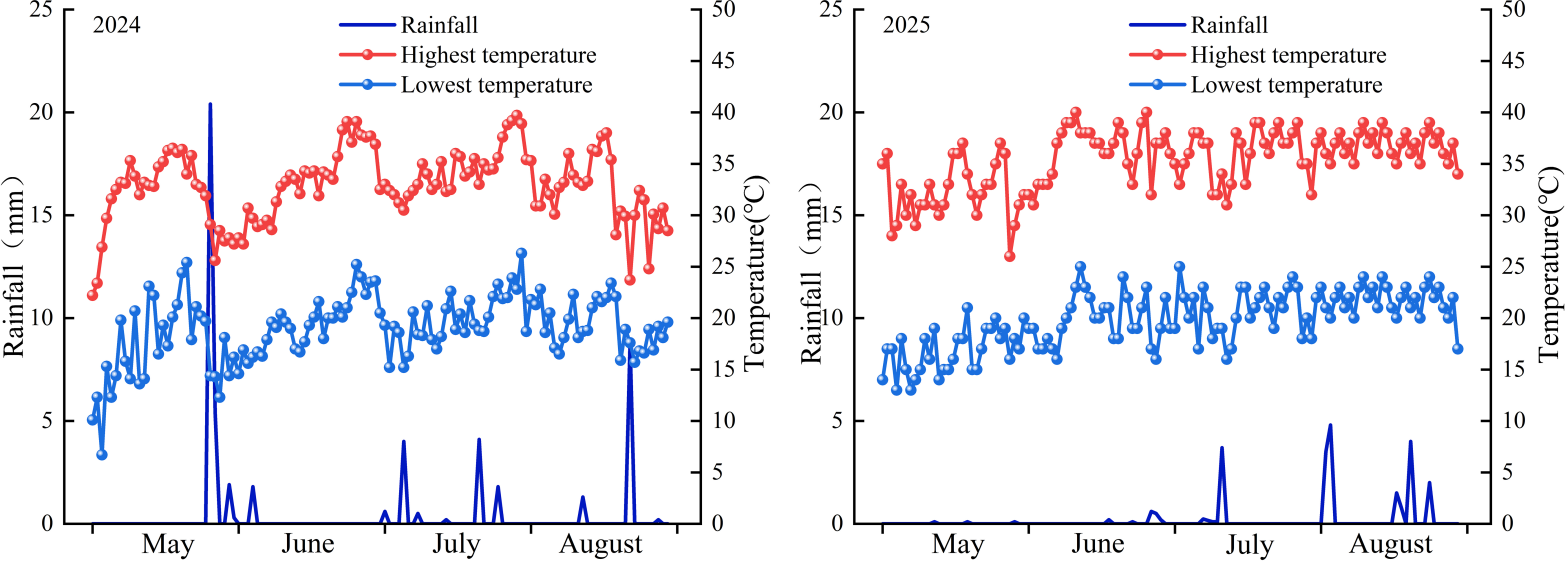
Rainfall and temperature at the experimental site in 2024–2025.

This study was conducted from 2024 to 2025 at the cotton experimental field in Alar City, Xinjiang Uygur Autonomous Region (40°56′N, 81°27′E). This region has a warm temperate, extreme continental arid desert climate, with an average annual temperature of 10.7 °C, annual precipitation ranging from 40.1-82.5 mm, and annual evaporation ranging from 1876–2559 mm. The area is part of the Tarim River alluvial fine soil plain, primarily composed of sandy loam soil (USDA soil taxonomy). The total nitrogen content is 0.68 g·kg^−1^, the total phosphorus content is 0.90 g·kg^−1^, the available phosphorus content is 37.94 mg·kg^−1^, the available potassium content is 160 mg·kg^−1^, and the organic matter content is 9.05 g·kg^−1^.Meteorological data for 2024 and 2025 are shown in **Figure 1**.

### 2.2 Experimental design

**Figure 2:**
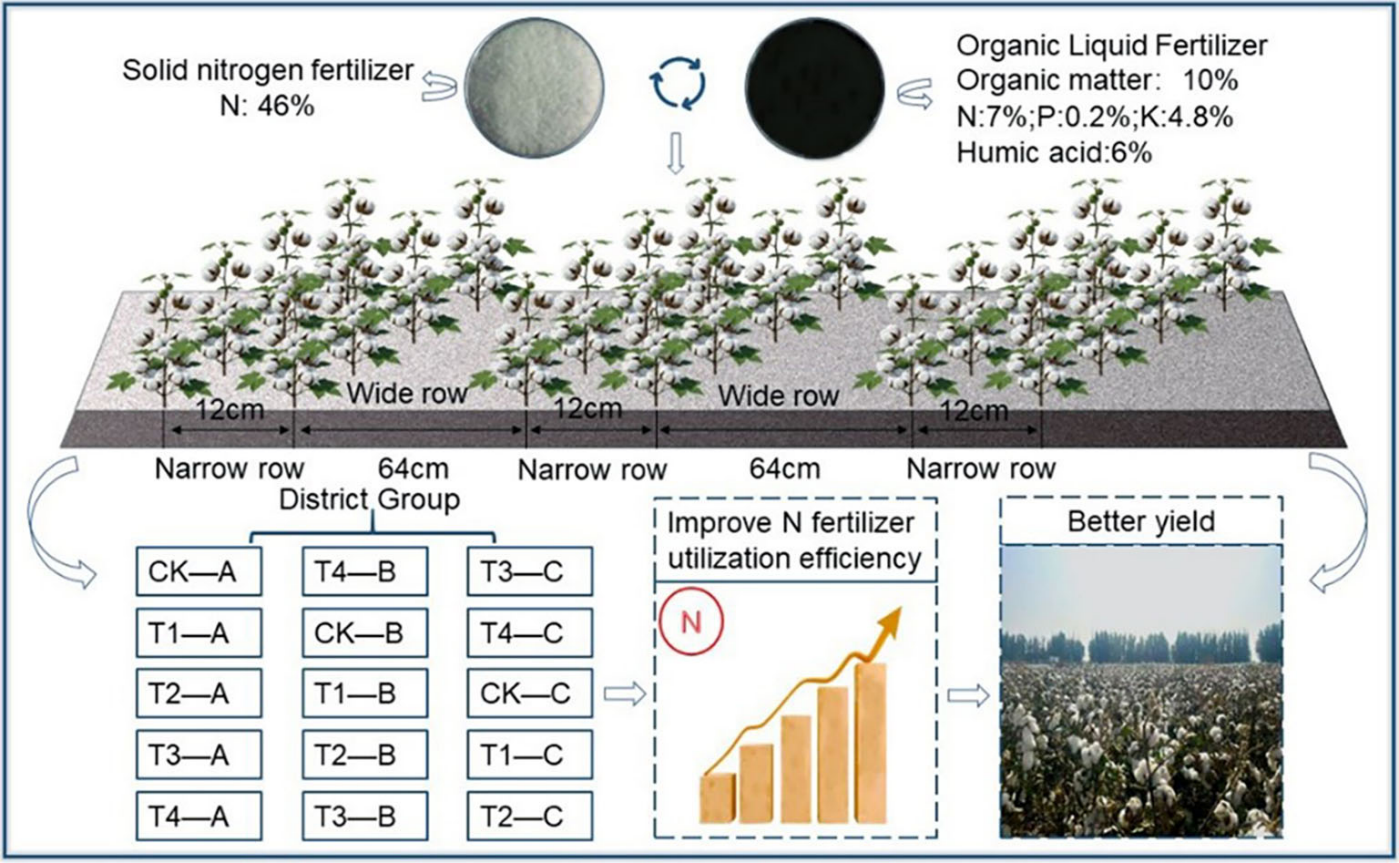
Schematic diagram of cotton planting.

The experiment was set up with five treatments: no-N control (CK), 100% urea (T1), and OLF replacing 15% (T2), 30% (T3), and 45% (T4) of urea-N. Each treatment was replicated three times; and arranged in a randomized block design, totaling 15 plots, each with an area of 96 m² (2.4 m wide × 40 m long). The cotton cultivation process is illustrated in **Figure 2**.

The nitrogen fertilizer used in the test was urea (N 46%), supplied by Xinjiang Yuxiang Huyang Chemical Co., Ltd.; the OLF (SOM 10%, N 7%, P_2_O_5_ 0.2%, K_2_O 4.8%, Humic acid 6%) was purchased from Shandong Minhe Biotechnology Co., Ltd.; the phosphorus fertilizer used in the test was superphosphate (containing P_2_O_5_ 32%). The potassium fertilizer used in the test was potassium sulfate (containing 52% K_2_O). The application rates of phosphorus and potassium fertilizers were identical across all treatments, with both applied as a single basal dressing. Nitrogen fertilizer was drip-applied in seven split applications during the squaring stage, peak squaring stage, initial flowering stage, peak flowering stage, boll setting stage, peak boll setting stage, and boll opening stage.The types and rates of fertilizers applied for each treatment are shown in **Table 1**.

**Table 1:** Types and amounts of fertilizers applied.

Processing	Fertilization treatment	Types and amounts of fertilizers
CK	blank	No nitrogen fertilizer applied
T1	100% CF	400 kg·ha^-1^ urea
T2	15% OLF+85% CF	300 kg·ha^-1^ urea and 394 kg·ha^-1^ organic liquid fertilizer
T3	30% OLF+70% CF	280 kg·ha-^1^ of urea and 789 kg·ha^-1^ of organic liquid fertilizer
T4	45% OLF+55% CF	220 kg·ha^-1^ of urea and 1183 kg·ha^-1^ of organic liquid fertilizer

CK represents no nitrogen fertilizer application, serving as a control; CF represents chemical nitrogen fertilizer (urea); OLF represents organic liquid fertilizer.

### 2.3 Plant sampling and analysis

During the seedling stage (SS), peak squaring stage (PSS), peak flowering stage (PFS), peak boll setting stage (PBS), and boll opening stage (BOS) of cotton, five representative plants with uniform growth were selected. The roots, stems, leaves, buds, flowers, and bolls were separated, blanched at 105 °C for 30 minutes, then dried at 80 °C until constant weight was achieved. After weighing, the dried samples were ground and sieved through a 0.5 mm sieve for later use.

### 2.4 Soil sampling and analysis

The test soil was naturally air-dried, ground, mixed uniformly, and sieved. Samples were collected once each during the SS, PSS, PFS, PBS, and BOS of cotton, totaling five samples over the entire growth period. Soil samples were taken from the 0–20 cm layer, divided into two portions, and placed in self-sealing bags. One portion was transferred to an ice box and stored at -20 °C for the determination of NH_4_^+^-N and NO_3_^--^N; The other soil sample is air-dried, ground, and sieved before nutrient content is determined. Soil total nitrogen(TN) is determined using the Kjeldahl nitrogen determination method; NO_3_^--^N is measured using the ultraviolet spectrophotometric method; NH_4_^+^-N is determined using the indigo carmine colorimetric method; alkali-hydrolyzed nitrogen(AH-N) content is determined using the alkali-hydrolyzed diffusion method; available phosphorus(AP) is determined using the 0.5 mol/L NaHCO_3_ method, with colorimetry at 880 nm using the molybdenum-antimony colorimetric method; available potassium(AK) content is determined using NHOAC extraction and flame photometry.

### 2.5 Cotton yield and yield components

The number of cotton plants per hectare can be quantified using two indicators: cotton row spacing (CRS)and cotton plant spacing (CPS).

CRS(m): Take 11 rows of cotton measurements at each sample point and calculate the average row spacing.

CPS(m): Randomly select 21 plants from one row at each sampling point to measure plant spacing and calculate the average plant spacing.

Number of plants per hectare (NPPH) = 10,000 m²/(CRS × CPS)

Cotton seed production is quantified by three indicators: number of trees per hectare (NPPH), average number of bolls per tree (NBPT), and weight per boll (WPB).

Cotton lint yield (kg·ha^-1^) = NPPH × NBPT × WPB/1000

### 2.6 Statistical analysis

One-way analysis of variance (ANOVA) was performed with SPSS 22.0 (IBM, Armonk, NY, USA) using the Duncan method. Significant differences were tested among treatments at the p < 0.05 probability level. All figures were drawn using Origin (Pro 2024, Origin Lab, Northampton, MA, USA).

## 3 Results

### 3.1 Dry matter distribution ratio of cotton

**Figure 3:**
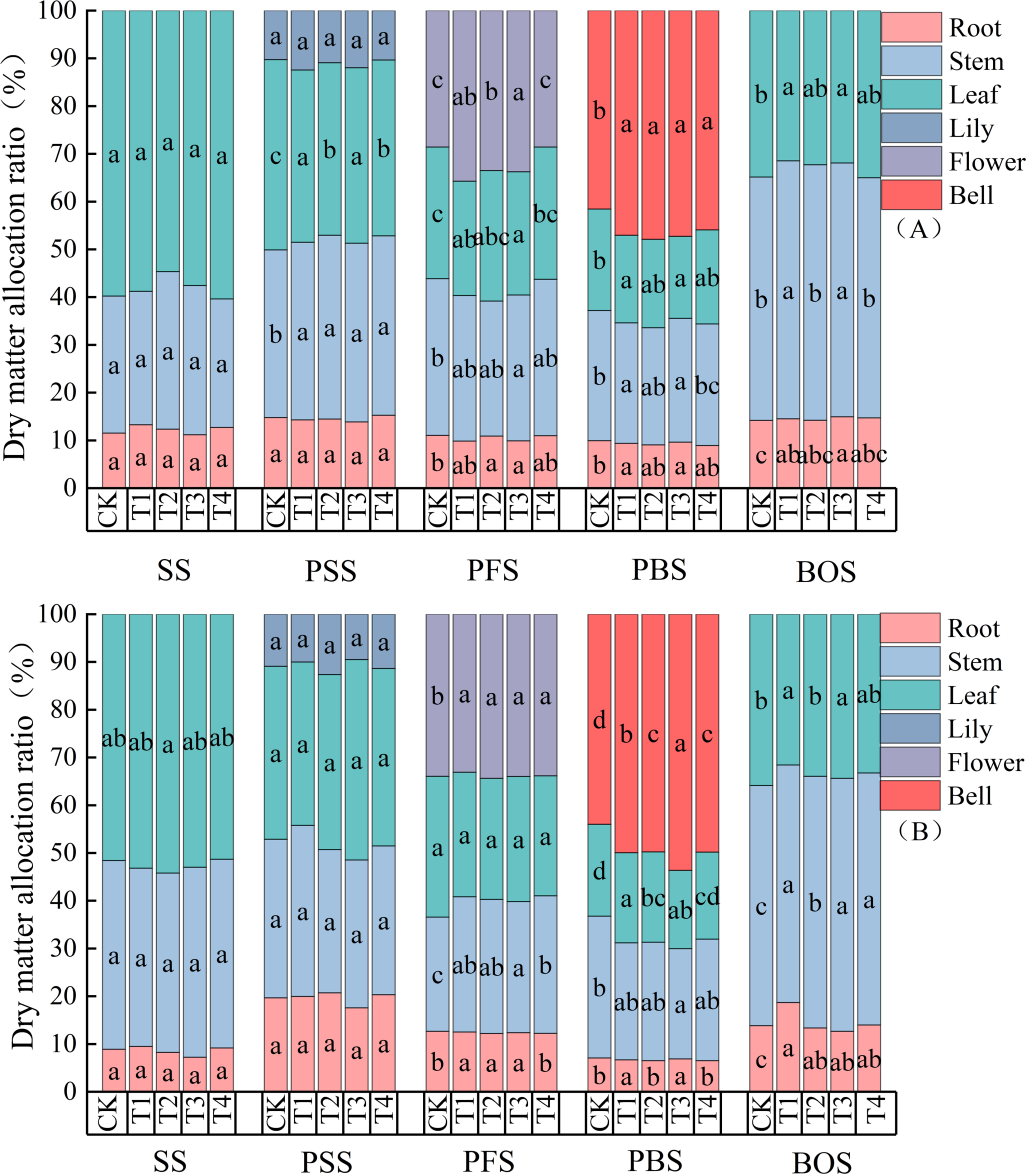
Dry Matter Distribution Ratio of Cotton. SS, seedling stage, PSS, peak squaring stage, PFS, peak flowering stage, PBS, peak boll setting stage, BOS, boll opening stage. CK, no nitrogen application; T1, conventional nitrogen application (100% urea); T2, 15% organic liquid fertilizer nitrogen + 85% urea nitrogen; T3, 30% organic liquid fertilizer nitrogen + 70% urea nitrogen; T4, 45% organic liquid fertilizer nitrogen + 55% urea nitrogen. A and B represent the dry matter allocation ratios for cotton in 2024 and 2025, respectively. Different letters indicate a significant difference among treatments at p < 0.05 by a Duncan test.

In 2024, The substitution of chemical fertilizers with organic liquid fertilizers at different growth stages had a minor effect on the dry matter allocation ratio between flowers and roots in cotton, but significantly influenced the allocation ratio between leaves and stems (**Figure 3**). The stem dry matter accounted for the highest proportion during the PSS stage. Both the PFS and PBS stages exhibited the highest dry matter allocation to flowers and bolls. The substitution of chemical fertilizers with organic liquid fertilizer during the PBS stage had a greater impact on the allocation ratio of dry matter between buds, flowers, and bolls than during the peak budding and peak flowering stages. As nitrogen application rates increased, the dry matter allocation ratio between leaves and stems showed a decreasing trend from the PSS to PBS periods, though the differences were small. The OF-30 treatment reduced the dry matter allocation ratio between leaves and stems by 19.6% and 8.8%, respectively. The bud-flower-boll dry matter allocation ratio gradually increased, with changes ranging from 10.3% to 31.3% for CK, 12.4% to 34.7% for U, 10.9% to 37.0% for OF-15, 12.0% to 35.3% for OF-30, and 10.3% to 35.6% for OF-30 (**Figure 3A**). In contrast, in 2025, the soil’s own nutrient reserves were sufficient, buffering fluctuations caused by different fertilization treatments and providing a foundation for high and stable cotton yields (**Figure 3B**).

### 3.2 Total nitrogen content in various organs of cotton plants

**Figure 4:**
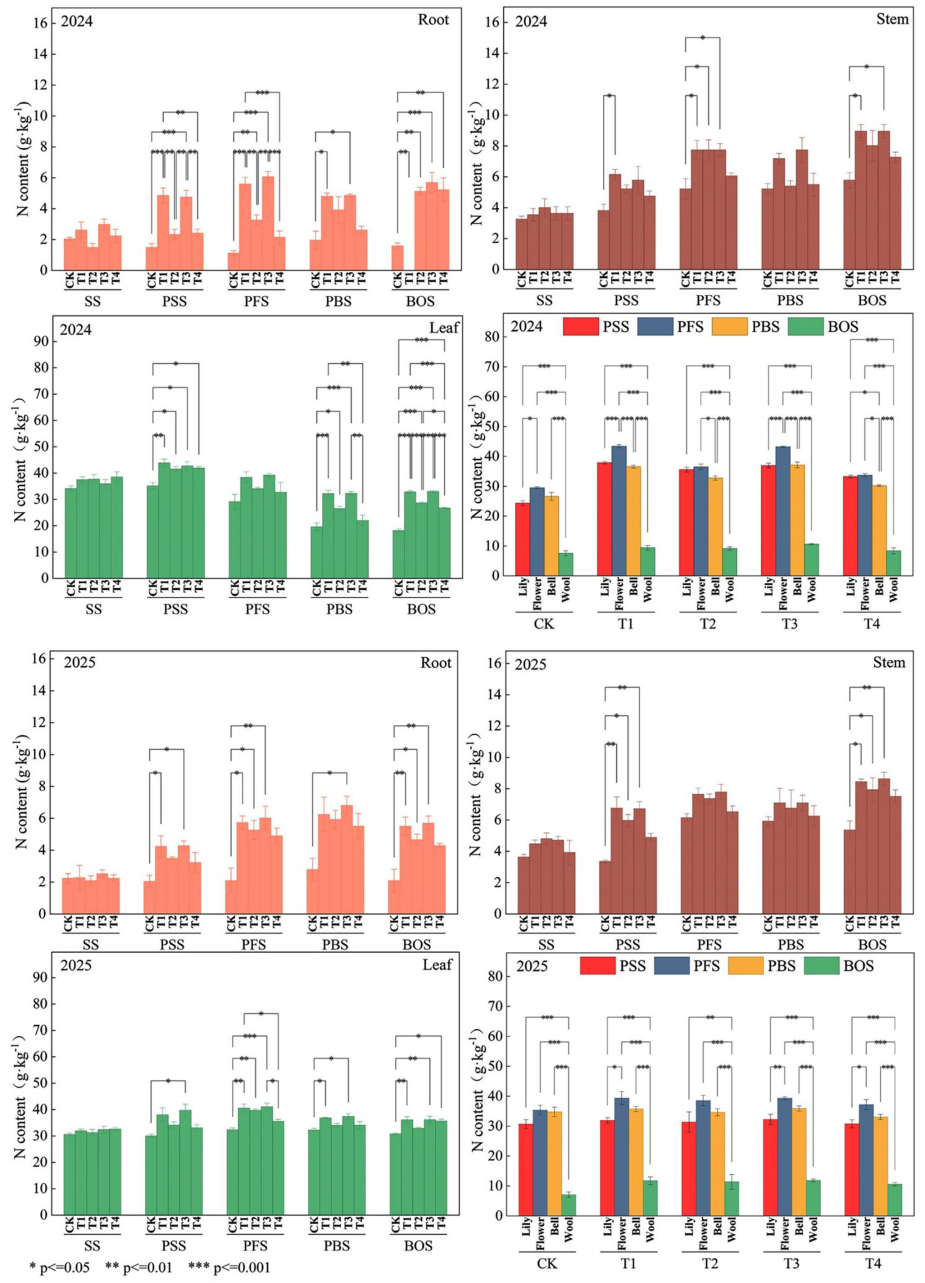
Total nitrogen content in various organs of cotton plants. SS, seedling stage, PSS, peak squaring stage, PFS, peak flowering stage, PBS, peak boll setting stage, BOS, boll opening stage. CK, no nitrogen application; T1, conventional nitrogen application (100% urea); T2, 15% organic liquid fertilizer nitrogen + 85% urea nitrogen; T3, 30% organic liquid fertilizer nitrogen + 70% urea nitrogen; T4, 45% organic liquid fertilizer nitrogen + 55% urea nitrogen. * means a significant difference at P < 0.05 level. ** means a significant difference at P < 0.01 level. *** means a significant difference at P<0.001 level.

In 2024, nitrogen content in all plant organs treated with nitrogen fertilizer exceeded that of the CK. During the SS stage, no significant differences in nitrogen content were observed among treatments in roots, stems, or leaves. During the PSS stage, the T1 and T3 treatment groups exhibited the highest nitrogen content in roots, stems, and leaves, significantly exceeding that of the CK. As nitrogen input increased, the nitrogen content in roots and leaves of the T3 treatment during the PFS period surpassed that of other treatments, reaching 6.07 and 39.2, respectively. However, stem nitrogen content stabilized and showed no significant differences among the T1, T2, and T3 treatments. During the PBS stage, total leaf nitrogen content declined rapidly. Compared to the earlier stage, CK, T1, T2, T3, and T4 treatments decreased by 32.7%, 16.1%, 22.2%, 17.6%, and 32.6%, respectively. During the BOS stage, nitrogen content in roots and stems of all fertilized treatments was significantly higher than CK, with no significant differences among fertilized treatments. Under T2 and T3 fertilization treatments, leaf nitrogen content showed no significant differences. However, T3 exhibited a 1.4% increase in nitrogen content compared to T2. Furthermore, T3 leaf nitrogen content was significantly higher than the control group, T2 group, and T4 group by 81.5%, 15.3%, and 23.8%, respectively (**Figure 4**-2024).

By 2025, the effects of each treatment on plant organs gradually became apparent as the growth period progressed. At SS, no significant differences were observed among treatments for roots, stems, or leaves. Upon entering PSS, treatments T1 and T3 showed significantly higher values than CK for roots, stems, and leaves, demonstrating superior promotion effects. By PFS, T1, T2, and T3 significantly outperformed CK for roots and leaves, with stems showing significant increases except for T4. No significant differences were observed in reproductive organs. In PBS, T3 showed the best performance for roots and leaves, while effects on stems and reproductive organs were insignificant across treatments. By BOS, all treatments significantly outperformed CK for roots, stems, and leaves.

During the two-year cotton cultivation period, cotton fiber exhibited nitrogen content below 10 g·kg^−^¹ across all treatments at different growth stages, while Flower, Cotton bud, and cotton boll all showed nitrogen content exceeding 20 g·kg^−^¹(**Figure 4**-2025).

### 3.3 Cotton lint yield

Two years of field trials showed that the fertilization treatment (P) significantly affected boll number per plant (NBPT), lint yield (CLY), agronomic nitrogen use efficiency (ANUE), and partial productivity of fertilizer nitrogen (PFP) (P < 0.01), but had no significant effect on weight per boll (WPB). Year (Y) exerted significant or extremely significant effects on NBPT, WPB, CLY, and PFP (**Table 2**). In 2024, the T3 treatment yielded the highest number of bolls, exceeding the nitrogen-free control (CK) and conventional fertilization treatment (T1) by 17.2% and 1.1%, respectively. The average boll weight of T1 and T3 treatments was essentially equivalent (5.64 g and 5.68 g, respectively), exceeding the CK treatment by 0.76 g and 0.72 g, respectively, with no significant differences among treatments. Cottonseed yield exhibited an initial increase followed by a decrease as the substitution ratio increased. The T3 treatment yielded the highest output, showing increases of 34%, 0.7%, 14.6%, and 18.6% compared to the control, T1, T2, and T4 treatments, respectively.

**Table 2:** Changes in cotton yield and yield components under different treatments.

Year	Processing	NBPT	WPB (g)	CLY (kg·ha^-1^)	ANUE (kg·kg^-1^)	PFP (kg·kg^-1^)
2024	CK	6.12a	4.92a	6248.8b	–	–
	T1	7.09a	5.64a	8309.7a	5.15a	20.77a
	T2	6.94a	5.13a	7308.1ab	2.65ab	18.27ab
	T3	7.17a	5.68a	8375.3a	5.32a	20.94a
	T4	6.12a	4.97a	7060.1b	2.23b	17.65b
2025	CK	7.29b	5.47a	8275.1b	–	–
	T1	7.98ab	5.76a	9554.1a	3.2a	23.89a
	T2	7.78ab	5.60a	9075.7ab	2.0ab	22.69a
	T3	8.22a	5.89a	10014a	4.34a	25.03a
	T4	7.72ab	5.57a	8905.6ab	1.57ab	22.26a
Processing(P)		**	ns	**	**	**
Year(Y)		*	*	**	ns	**
P*Y		ns	ns	ns	ns	*

NBPT, number of bolls per tree, WPB, weight per boll, ANUE, Agronomic Nitrogen Use Efficiency, PFP, Partial Factor Productivity of Nitrogen. CK, no nitrogen application; T1, conventional nitrogen application (100% urea); T2, 15% organic liquid fertilizer nitrogen + 85% urea nitrogen; T3, 30% organic liquid fertilizer nitrogen + 70% urea nitrogen; T4, 45% organic liquid fertilizer nitrogen + 55% urea nitrogen. Data are shown in mean value (n = 3). Different letters indicate a significant difference among treatments at p < 0.05 by a Duncan test. *means a significant difference at P < 0.05 level. **means a significant difference at P < 0.01 level, while ns means no significant difference.

In 2025, T3 treatment maintained the highest NBPT yield, exceeding CK and T1 treatments by 12.8% and 3.0%, respectively. CLY treatment increased yields by 21.0% and 4.81% compared to CK and T1 treatments, respectively. T3 treatment exhibited significantly higher ANUE than other treatments, reaching 1.36 times, 2.17 times, and 2.76 times that of T1, T2, and T4 treatments, respectively.

### 3.4 Nitrogen content in different soil forms

**Figure 5:**
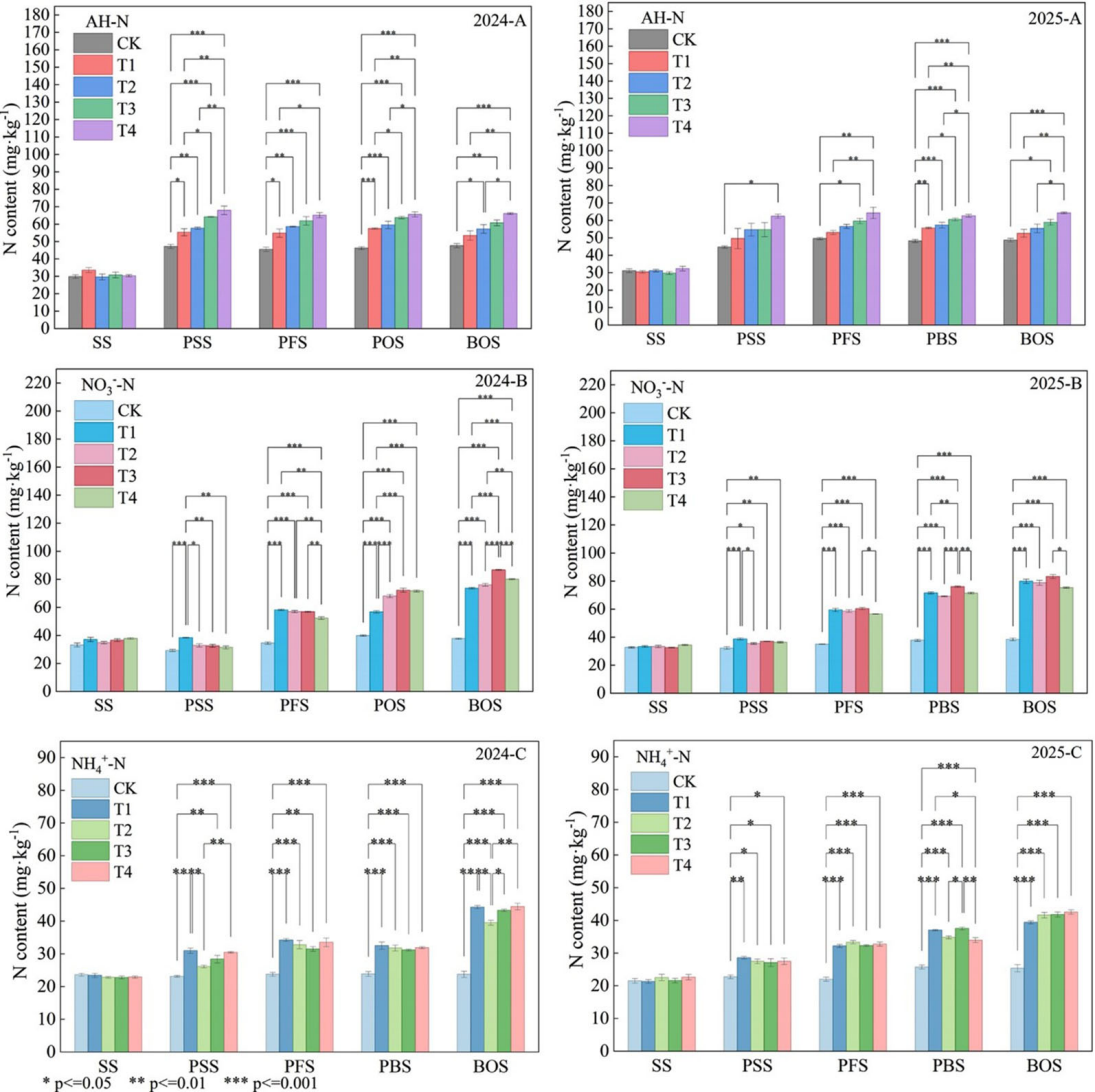
Nitrogen content in different forms in soil at 0–20 cm depth during different stages of growth, SS, seedling stage, PSS, peak squaring stage, PFS, peak flowering stage, PBS, peak boll setting stage, BOS, boll opening stage, AH-N, Alkali-Hydrolyzable Nitrogen, NH4+-N, soil Ammonium Nitrogen, NO3--N, Soil Nitrate Nitrogen, CK, no nitrogen application; T1, conventional nitrogen application (100% urea); T2, 15% organic liquid fertilizer nitrogen + 85% urea nitrogen; T3, 30% organic liquid fertilizer nitrogen + 70% urea nitrogen; T4, 45% organic liquid fertilizer nitrogen + 55% urea nitrogen. * means a significant difference at P < 0.05 level. ** means a significant difference at P < 0.01 level. *** means a significant difference at P<0.001 level.

The use of OLF as a substitute for chemical fertilizer significantly increased the AH-N content at different growth stages. During the PFS, the AH-N content in the substitute treatments T3 and T4 was the highest, significantly higher than that in other treatments, at 64.17 mg·kg^−^¹ and 67.9 mg·kg^−^¹ respectively; During the PFS, the AH-N content in the T4 treatment was the most significant, at 65.1 mg·kg^−^¹, which was 43.1% higher than the CK treatment without fertilization and 18.7% higher than the T1 treatment with pure nitrogen fertilizer. During the PBS, there was no significant difference between the T3 and T4 treatments, but both were significantly higher than the other treatments. During the BOS, the AH-N content of the T4 treatment was 15% and 8.8% higher than that of the T2 and T3 treatments, respectively. The results indicate that a 45% OLF replacement ratio has the most stable effect on increasing AH-N (**Figure 5**-2024-A). During the SS stage of the 2025 planting season, no significant differences in AH-N content were observed among treatments. Starting from the PSS stage, treatment differences began to emerge, with the T4 treatment (45% substitution) exhibiting the highest AH-N content at 62.42 mg·kg^−^¹, significantly exceeding that of the CK treatment. This advantage was consistently maintained throughout subsequent PFS, PBS, and BOS stages. The T4 treatment exhibited significantly higher AH-N content than CK at all fertilization periods, peaking at 64.28 mg·kg^−^¹ during the boll opening stage— the highest among all treatments (**Figure 5**-2025-A).

As nitrogen application rates increased, NO_3_^−^-N content gradually increased in the 0–20 cm soil layer across all fertilization treatments. During the PSS and PFS, the order was T1 > T2 > T3 > T4 > CK, while during the PFS and BOS, the order was T3 > T4 > T2 > T1 > CK. NO_3_^−^-N content reached its highest levels during the BOS, with significant differences, ranging from 73.55 mg·kg^−^¹ to 86.65 mg·kg^−^¹. The T3 treatment had the highest NO_3_^−^-N content in the soil, at 86.65 mg·kg-1, significantly higher than the CK and T1 treatments, with increases of 130.20% and 17.81%, respectively; Secondly, the T2 and T4 treatments were significantly higher than the CK and T1 treatments, exceeding the CK by 102.05% and 112.61%, respectively, and exceeding the T1 treatment by 3.40% and 8.80%, respectively (**Figure 5**-2024-B). In 2025, during the PSS and PFS stages, NO_3_^−^-N content began to rise significantly, with T1 treatment exhibiting the highest levels at this point. Chemical nitrogen fertilizers demonstrated a faster ability to elevate soil nitrogen levels in the early stages. During the PBS and BOS periods, soil NO_3_^−^-N content reached its peak. The T3 treatment recorded NO_3_^−^-N levels of 75.94 mg·kg^−^¹ and 83.25 mg·kg^−^¹ during the PBS and BOS periods, respectively, both significantly higher than all other treatments. This indicates that the nutrient release pattern of the T3 treatment better aligns with cotton’s nutrient demand patterns, enabling sustained soil nitrogen supply throughout the mid-to-late growth stages (**Figure 5**-2025-B).

The NH_4_^+^-N content in the 0–20 cm soil layer of all treatments remained relatively stable before the PBS and slightly increased during the BOS. During the BOS, the NH_4_^+^-N content ranged from 39.53 mg·kg^−^¹ to 44.47 mg·kg^−^¹ across all fertilization treatments, with the order of T4 > T3 > T1 > T2. However, the differences between T1, T3, and T4 were not significant. All fertilization treatments were significantly higher than the CK treatment. The T4 treatment was 87.21% higher than the CK treatment, 0.43% higher than the T1 treatment, and 12.50% and 2.63% higher than the T2 and T3 treatments, respectively (**Figure 5**-2024-C). In 2025, during the PBS period, NH_4_^+^-N content exhibited significant differences, with T1 and T3 treatments showing the highest levels at 37.02 and 37.54 mg·kg^−^¹, respectively, which were significantly higher than those in T2 and T4 treatments (**Figure 5**-2025-C).

### 3.5 BOS period 0–20 Nitrogen content in different forms in soil and percentage of total nitrogen

The partial replacement of chemical fertilizers with OLF had a significant impact on soil TN content (**Table 3**). The T3 treatment had the highest TN content at 0.66 g·kg^-1^, while the T2 treatment had the lowest at 0.42 g·kg^-1^. Compared to the CK treatment without fertilization, all fertilization treatments significantly increased TN content, with increases ranging from 0.22 g·kg^−^¹ to 0.46 g·kg^−^¹. The overall trend showed T3 > T4 > T2 > T1 > CK, indicating that a higher replacement ratio of organic liquid fertilizer is not necessarily better, and a 30% replacement ratio yields the optimal effect on soil total nitrogen content. In terms of nitrogen form composition, the primary available nitrogen form in the surface soil of all replacement treatments was NO_3_^--^N, accounting for 12.06–18.11%, while AH-N and NH_4_^+^-N accounted for relatively lower proportions (**Table 3**).

**Table 3:** BOS period 0-20 Nitrogen content in different forms in soil and percentage of total nitrogen.

Year	Processing	Nitrogen content in various forms				Percentage of total nitrogen (%)		
		TN (g·kg^-1^)	AH-N (mg·kg^-1^)	NO_3_^-^-N (mg·kg^-1^)	NH_4_^+^-N (mg·kg^-1^)	AH-N	NO_3_^-^-N	NH_4_^+^-N
2024	CK	0.20c	47.60e	37.64e	23.75c	23.80	18.82	11.88
	T1	0.61a	53.43cd	73.55d	44.28a	8.76	12.06	7.26
	T2	0.42b	57.17bc	76.05c	39.53ba	13.61	18.11	9.41
	T3	0.66a	60.67ab	86.65a	43.33a	9.19	13.13	6.57
	T4	0.56ab	66.03a	80.03d	44.47a	11.79	14.29	7.94
2025	CK	0.34b	48.65d	38.34c	25.39c	14.31	11.28	7.74
	T1	0.51a	52.62cd	79.79b	39.41b	10.32	15.65	7.73
	T2	0.45b	55.42bc	78.73ab	41.63ab	12.32	17.50	9.25
	T3	0.54a	58.92ab	83.25ab	41.80ab	10.91	15.42	7.74
	T4	0.50a	64.28a	75.28a	42.56a	12.86	15.06	8.51
Processing(P)		***	***	***	**	–	–	–
Year(Y)		ns	ns	ns	ns	–	–	–
P*Y		**	ns	***	**	–	–	–

Nitrogen content in different forms in soil at 0–20 cm depth during different stages of growth, TN, Total Nitrogen, AH-N, Alkali-Hydrolyzable Nitrogen, NH4+-N, soil Ammonium Nitrogen, NO3--N, Soil Nitrate Nitrogen, CK, no nitrogen application; T1, conventional nitrogen application (100% urea); T2, 15% organic liquid fertilizer nitrogen + 85% urea nitrogen; T3, 30% organic liquid fertilizer nitrogen + 70% urea nitrogen; T4, 45% organic liquid fertilizer nitrogen + 55% urea nitrogen. Different letters indicate a significant difference among treatments at p < 0.05 by a Duncan test. *means a significant difference at P < 0.05 level. ** means a significant difference at P < 0.01 level. *** means a significant difference at P<0.001 level, while ns means no significant difference.

Consistent with the 2024 trend, TN content in all fertilized treatments in 2025 was significantly higher than in the nitrogen-free CK treatment. Among these, the T3 treatment exhibited the highest TN content at 0.54 g·kg^−^¹, comparable to the levels observed in T1 and T4 treatments. NO_3_^−^-N remained the predominant available nitrogen form in the soil, with T3 treatment maintaining the highest nitrate nitrogen content in 2025. NH_4_^+^-N were highest in the T4 treatment. Regarding the percentage of available nitrogen forms relative to total nitrogen (TN), NO_3_^−^-N accounted for 11.28%–17.50%, generally exceeding the proportion of NH_4_^+^-N. Among these, the T2 treatment exhibited the highest nitrate nitrogen proportion in 2025, reaching 17.50% (**Table 3**).

### 3.6 Soil available potassium

**Figure 6:**
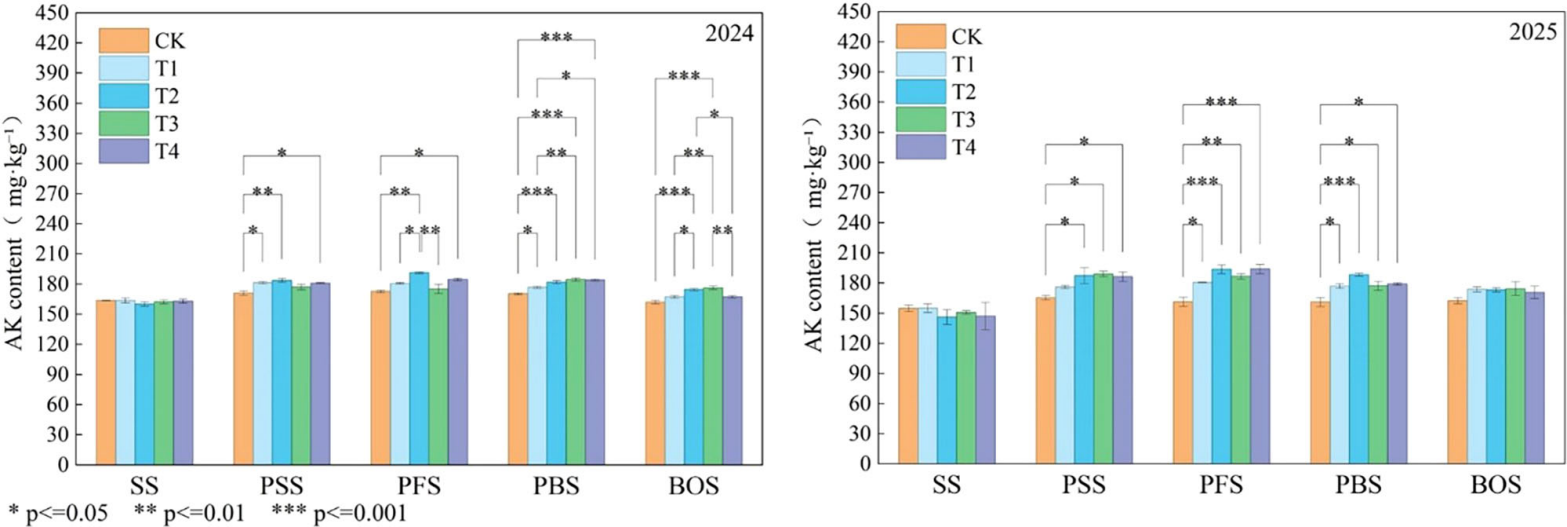
The effect of organic liquid fertilizer replacement of chemical fertilizer on soil available potassium, SS, seedling stage, PSS, peak squaring stage, PFS, peak flowering stage, PBS, peak boll setting stage, BOS, boll opening stage, CK, no nitrogen application; T1, conventional nitrogen application (100% urea); T2, 15% organic liquid fertilizer nitrogen + 85% urea nitrogen; T3, 30% organic liquid fertilizer nitrogen + 70% urea nitrogen; T4, 45% organic liquid fertilizer nitrogen + 55% urea nitrogen * means a significant difference at P < 0.05 level. ** means a significant difference at P < 0.01 level. *** means a significant difference at P<0.001 level.

The average content of AK in the treatments using OLF as a partial replacement for chemical fertilizer ranged from 162 mg·kg^−^¹ to 191.33 mg·kg^−^¹. During the PSS and PFS, the T2 treatment had the highest AK content, at 183.67 mg·kg^−^¹ and 191.33 mg·kg^−^¹, respectively. During the PBS and BOS, the T3 treatment had the highest AK content, at 184.67 mg·kg^−^¹ and 176.33 mg·kg^−^¹, respectively; The AK content of each alternative treatment differed significantly from the no-fertilizer treatment. When OLF replaced 30% of chemical fertilizer, it contributed more to AK than conventional fertilization. During the BOS, the order was T3 > T2 > T4 > T1 > CK, indicating that a 30% OLF replacement ratio had the optimal effect on increasing AK. (**Figure 6**-2024). Compared to 2024, the increase in AK content among different fertilization treatments in 2025 was relatively moderate, with smaller differences between treatments. Throughout the entire growth period, AK content reached higher levels in the PSS and PFS treatments. During the PBS stage, only the T2 treatment showed significantly higher AK content than the CK treatment, while T1, T3, and T4 treatments exhibited no significant differences compared to CK. This contrasts with the 2024 results, where multiple alternative treatments demonstrated significant enhancement effects (**Figure 6**-2025).

### 3.7 Soil available phosphorus

**Figure 7:**
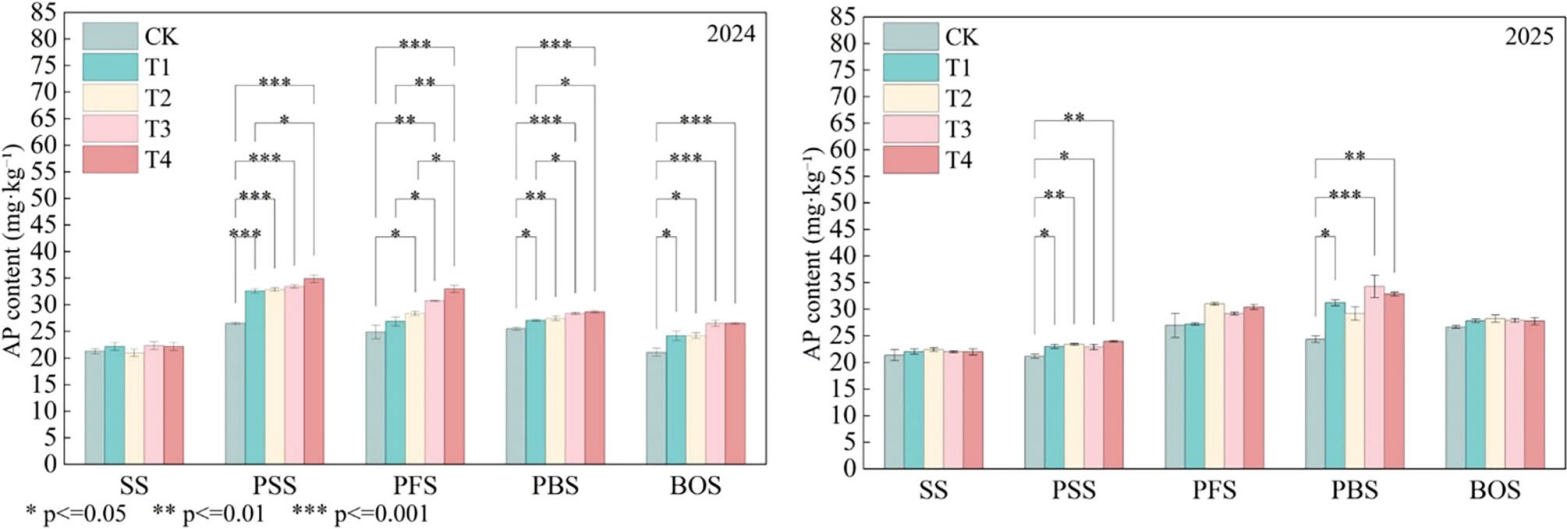
The effect of organic liquid fertilizer replacement of chemical fertilizer on soil available potassium, SS, seedling stage, PSS, peak squaring stage, PFS, peak flowering stage, PBS, peak boll setting stage, BOS, boll opening stage, CK, no nitrogen application; T1, conventional nitrogen application (100% urea); T2, 15% organic liquid fertilizer nitrogen + 85% urea nitrogen; T3, 30% organic liquid fertilizer nitrogen + 70% urea nitrogen; T4, 45% organic liquid fertilizer nitrogen + 55% urea nitrogen * means a significant difference at P < 0.05 level. ** means a significant difference at P < 0.01 level. *** means a significant difference at P<0.001 level.

The AP content in each treatment was T4 > T3 > T2 > T1 > CK, with average values ranging from 20.98 mg·kg^−^¹ to 34.93 mg·kg^−^¹. Compared to CK and T1, the increase in T4 treatment was 6.0% and 4.53%, respectively; During the entire growth period, the AP content was highest in the T4 treatment, reaching a peak of 33.01 mg·kg^−^¹ during the PFS. Among the fertilization treatments, the AP content was highest during the PFS, with increases of 7.90%, 14.02%, 23.51%, and 32.46% compared to the untreated control; Compared with conventional fertilization, the alternative treatments showed increases of 5.67%, 14.46%, and 22.76%, respectively. There were no significant differences among the T2, T3, and T4 treatments, but all were significantly higher than the CK and T1 treatments. (**Figure 7**-2024).

In 2025, during the SS and PSS periods, there were no significant differences in AP content among fertilizationer treatments. However, starting from the PFS period, AP content significantly increased and treatment differences were also enhanced. At PFS, treatment T2 exhibited the highest AP content, reaching 31.08 mg·kg^−^¹. After the PBS period, treatment T3 achieved the peak AP content throughout the entire growth period at 34.31 mg·kg^−^¹, which was significantly higher than that of the CK treatment (**Figure 7**-2025).

### 3.8 Correlation analysis of soil nutrients and yield components on seed cotton yield

**Figure 8:**
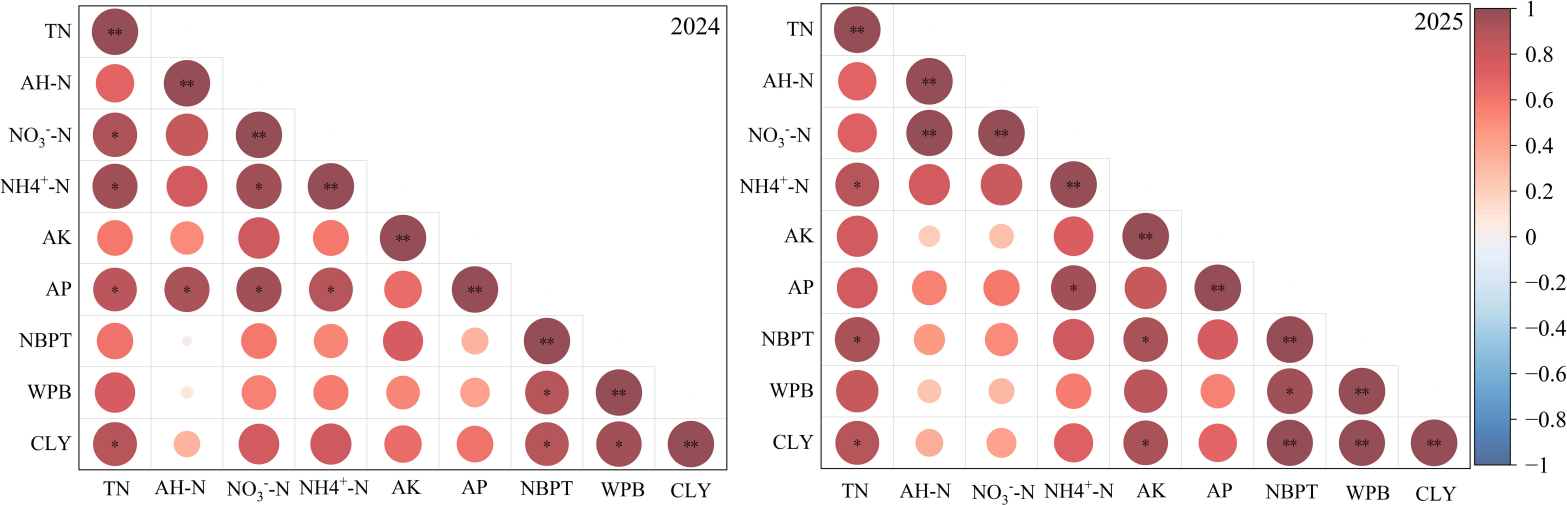
Correlation Analysis of Soil Nutrients and Seed Cotton Yield Factors, TN, Total Nitrogen, AH-N, Alkaline Hydrolysis Nitrogen, NH_4_^+^-N, Soil Ammonium Nitrogen, NO_3_^−^-N,Soil Nitrate Nitrogen, AP,Soil Available Phosphorus, AK, Soil Available Potassium, NBPT, number of bolls per tree, WPB, weight per. * means a significant difference at P < 0.05 level. ** means a significant difference at P < 0.01 level.

Analysis of the correlation between soil nutrients, yield components, and cotton lint yield (CLY) reveals that NO_3_^−^-N content and NBPT are the two most critical factors directly determining cotton yield. Both exhibit extremely strong positive correlations with CLY, while boll weight per boll (WPB) and other readily available soil nutrients such as AP and AK contribute relatively less. Therefore, ensuring adequate soil nitrate nitrogen supply during critical stages like the flowering and boll setting period through 30% organic nitrogen substitution, while promoting boll formation, represents an ideal pathway to achieve high-yield and efficient cotton production (**Figure 8**).

## 4 Discussion

### 4.1 Synergistic effects of organic-inorganic substitution on plant nitrogen uptake and yield

Nitrogen is one of the key nutrients for crop growth and yield formation (Wang et al., 2025; Zhao et al., 2025). Plant nitrogen uptake increases with higher nitrogen fertilizer application rates (Xu et al., 2024). In this study, treatment T3 achieved the highest CLY, ANUE, and PFP in both years. Compared to the pure chemical fertilizer treatment (T1), treatment T3 exhibited higher nitrogen content in both roots and leaves from PFS to PBS than other treatments (**Figure 4**). This indicates that the slow-release nutrients in organic liquid fertilizer complemented the fast-release nitrogen from urea effectively (Fan et al., 2025). Urea rapidly supplied nitrogen in the early stage, meeting cotton’s vegetative growth needs (Fan et al., 2025; Mathenge et al., 2019). Meanwhile, organic liquid fertilizers continuously release nitrogen through microbial decomposition and may promote plant growth and development via bioactive substances such as humic acids and amino acids. This enhances the nutrient absorption and assimilation capacity of the aboveground parts, thereby prolonging the fertilizer’s efficacy (Lu, 2020). Particularly during cotton’s peak nitrogen demand periods (PFS and PBS), this dual-release fertilization model prevented nitrogen supply gaps and premature senescence that might occur in pure chemical fertilizer treatments. It ensured boll development and dry matter accumulation, ultimately resulting in significantly increased net boll production (NBPT) (**Table 3**). This aligns with previous findings that combined organic-inorganic fertilization coordinates nutrient release to meet critical crop nutrient requirements. However, when the substitution ratio exceeded 45% (T4), both yield and nitrogen use efficiency declined. This likely resulted from the higher C/N ratio introduced by organic inputs triggering temporary microbial fixation of mineral nitrogen (Kong et al., 2011; Lopes et al., 2022). Consequently, a brief period of nitrogen competition occurred during the cotton’s rapid growth phase, which was detrimental to achieving high yields.

### 4.2 Effects of organic liquid fertilizer replacement on soil nitrogen forms

Soil nitrogen is a key factor in achieving high-yield, high-quality, and sustainable agricultural production (Mittermayer et al., 2024; Zhang et al., 2022). Research indicates that the combined application of organic and inorganic fertilizers promotes nutrient accumulation, effectively increasing soil organic matter, total nitrogen, and ammonium nitrogen content, thereby laying the foundation for high crop yields (Bhardwaj et al., 2025). Furthermore, organic fertilizer application effectively increases soil organic nitrogen reserves while elevating NH_4_^+^-N and NO_3_^−^-N concentrations, thereby enhancing soil nitrogen supply and achieving efficient coupling with crop nitrogen demand (Wang et al., 2023b). This experiment indicates that treatment T3 most effectively increased total nitrogen content, followed by treatment T4. This may result from the slower nitrogen release from OLF compared to the rapid nitrogen supply from chemical fertilizers (Feng et al., 2021). Combining both can balance nitrogen supply and demand. However, excessively high substitution ratios may lead to insufficient nitrogen supply, thereby reducing total nitrogen content (Zhou et al., 2023). As the OLF substitution ratio increased, soil available nitrogen content showed an upward trend. Across all treatment groups in the 0–20 cm soil layer, nitrogen primarily existed as NO_3_^−^-N, with NH_4_^+^-N content generally low. This likely resulted from OLF application increasing the soil carbon-to-nitrogen ratio, thereby promoting microbial mineralization of organic nitrogen and elevating soil NH_4_^+^-N content. High organic matter content suppressed nitrifying bacterial activity and enhanced NH_4_^+^-N adsorption, thereby exerting the optimal effect on NH_4_^+^-N content.

### 4.3 The effect of replacing part of chemical fertilizer with organic liquid fertilizer on readily AP and AK

Numerous studies indicate that the combined application of organic liquid fertilizers and chemical fertilizers can significantly increase the content of available phosphorus (AP) and available potassium (AK) in soil (Niu et al., 2022; Shi et al., 2023; Zeng et al., 2025). Relevant field trials confirm that under organic-inorganic composite application systems, phosphate increases during the current crop season can reach 15.43%–149.3%, while phosphate increases range from 7.75%–31.4%, with phosphate increases being particularly pronounced (Jing et al., 2022). The substitution of organic liquid fertilizers not only optimizes nitrogen cycling but also produces a significant synergistic enhancement effect on the availability of soil phosphorus and potassium nutrients (Wang et al., 2023c). The T3 treatment exhibited the highest soil available potassium (AK) content (184.67 mg·kg^−^¹) during the PBS (**Figure 5**). Furthermore, the available phosphorus (AP) content in all substitution treatments was significantly higher than that in the CK and T1 treatments, showing an upward trend with increasing substitution ratios (**Figure 6**). This phenomenon may be attributed to organic components in the organic liquid fertilizer, such as humic acids and amino acids, which can chelate calcium, iron, and aluminum ions in the soil, thereby desorbing and activating immobilized phosphorus (Xing et al., 2025). Additionally, functional groups on humic acid molecules can occupy adsorption sites for soil potassium, reducing potassium immobilization and promoting the release of adsorbed potassium (Teressa et al., 2024). Furthermore, various organic acids (e.g., citric acid, oxalic acid) produced during microbial decomposition of organic matter lower the pH in the rhizosphere, further dissolving insoluble phosphates (Teressa et al., 2024). This comprehensive nutrient activation capability— is unattainable with chemical fertilizers alone. That’s the key reason why organic-inorganic fertilizer combinations achieve “reduced fertilizer use with increased efficiency” and enhance soil fertility.

## 5 Conclusions

Based on a systematic evaluation of two years of field trials, this study conclusively demonstrates that replacing 30% of chemical fertilizer nitrogen with organic liquid fertilizer. Nitrogen represents the optimal fertilization strategy for drip-irrigated cotton fields in Xinjiang’s extreme arid regions. Treatment T3 achieved soil NO_3_^−^-N levels as high as 86.65 and 83.25 mg·kg^−1^ during boll setting (PBS), significantly optimizing soil nitrogen supply during the pre-flowering stage (PFS) and pre-boll setting stage (PBS). This enhanced cotton nitrogen uptake and boll set per plant, leading to a marked increase in lint yield (8375.3 and 10014 kg·ha^−1^ over two years, respectively). Concurrently, this substitution ratio effectively enhanced soil phosphorus and potassium nutrients, demonstrating synergistic effects between organic-inorganic combined application in improving soil fertility, boosting cotton yields, and achieving efficient resource utilization. This provides a reliable technical model and scientific basis for reducing chemical fertilizer application and promoting green production in drip-irrigated cotton fields in southern Xinjiang.

## Data Availability

The original contributions presented in the study are included in the article/supplementary material. Further inquiries can be directed to the corresponding authors.
