# Correction: Enhanced nitrogen dynamics and uptake in cotton via organic liquid fertilizer partial substitution

**DOI:** 10.3389/fpls.2026.1874796

**Published:** 2026-05-18

**Authors:** Frontiers Production Office

**Affiliations:** Frontiers Media SA, Lausanne, Switzerland

**Keywords:** chemical fertilizer, cotton, nitrogen utilization, organic liquid fertilizer, soil nutrients

Affiliation 1 was incorrectly presented as “Northwest A&F University Institute of Soil and Water Conservation, College of Agriculture, Tarim University, Alar, China”. The correction Affiliation 1 is “College of Agriculture, Tarim University, Alar, China”.

The original version of this article has been updated.

